# Reduced dislocation rate after hip arthroplasty for femoral neck fractures when changing from posterolateral to anterolateral approach: A prospective study of 372 hips

**DOI:** 10.3109/17453674.2010.519170

**Published:** 2010-10-08

**Authors:** Olof Sköldenberg, Anna Ekman, Mats Salemyr, Henrik Bodén

**Affiliations:** Karolinska Institutet, Department of Clinical Sciences at Danderyd Hospital, Stockholm, Sweden.

## Abstract

**Background and purpose:**

Recent studies have shown that compared to the posterolateral approach, the anterolateral approach reduces the risk of dislocation after hip arthroplasty in patients with femoral neck fractures. We have therefore started to use the anterolateral approach on these patients and we now report the consequences of this change for the dislocation rate.

**Patients and methods:**

We chose two 1-year time periods, 2007 (n = 199) and 2008 (n = 173), the former being before and the latter after the implementation of the anterolateral approach as the standard incision for hip arthroplasties in patients with femoral neck fractures. During 2007, 77% of the hips were operated on with the posterolateral approach and in 2008, 78% of the hips were operated on using the anterolateral approach.

**Results:**

The dislocation rate was reduced from 8% (16/199) in 2007 to 2% (3/173) in 2008. A multivariable logistic regression analysis showed that the posterolateral approach was the only factor associated with an increased risk of dislocation, with an odds ratio of 8 (2–35). Age, sex, ASA classification, type of arthroplasty, cognitive dysfunction, or the experience of the surgeon had no effect on the risk of dislocation.

**Interpretation:**

Since most of our surgeons had earlier used the posterolateral approach when performing hip arthroplasties in patients with a femoral neck fracture, this study shows our surgical learning curve. We conclude that a collective policy change regarding surgical approach for these patients is both feasible and to be recommended, as it leads to a substantial reduction in dislocation rate.

In Sweden, primary hip arthroplasty is now the routine treatment for elderly patients with a displaced femoral neck fracture. This is based on a number of studies showing better hip function, better quality of life, and a lower proportion of reoperations when compared to internal fixation (Johansson et al. 2000, Neander 2000, Tidermark et al. 2003, Baker et al. 2006, Rogmark and Johnell 2006). Dislocation after hip arthroplasty is a feared complication that can lead to reoperations as well as reduction in quality of life and functional outcome. Recently, 2 studies have shown that compared to the posterolateral approach (Moore 1957), the modified anterolateral approach (Gammer 1985) reduces the risk of dislocation after hemiarthroplasty (HA) and total hip arthroplasty (THA) in patients with femoral neck fractures (Enocson et al. 2008, 2009a).

At our institution, most surgeons were using the posterolateral approach for all hip arthroplasties. In 2008, on the basis of reported experience from other institutions, we changed to the anterolateral approach in patients with a femoral neck fracture. We now report what this change has led to with regard to dislocation rate and reoperations.

## Patients and methods

This study was conducted between January 2007 and December 2008 at the Orthopedics Department of Danderyd Hospital, Stockholm, Sweden. During that time, 372 consecutive hemiarthroplasties (HAs) and total hip arthroplasties (THAs) were performed on 368 patients (273 females) with a femoral neck fracture and they were included in a prospective cohort study (Table 1). The indication for surgery was a non-pathological displaced femoral neck fracture (Garden III or IV) (n = 309) or failed internal fixation after a femoral neck fracture (n = 63).

**Table 1.** Data on all hips included in the study

Characteristics	2007 (n = 199)	2008 (n = 173)	p-value
Age **^a^**	81 (8)	82 (8)	0.2
Sex **^b^**			
Female	145 (73)	132 (76)	0.5
Male	54 (27)	41 (24)	
Weight (kg) **^a^**	64 (13)	64 (14)	1.0
Height (cm) **^a^**	168 (8)	166 (9)	0.2
ASA-category **^b^**			0.3
ASA 1 or 2	106 (53)	80 (36)	0.2
ASA 3 or 4	93 (47)	93 (64)	
Cognitive dysfunction **^b^**			
No	156 (78)	132 (68)	0.8
Probable	16 (11)	17 (13)	
Certain	27 (11)	24 (19)	
Surgical approach **^b^**			
Anterolateral	46 (23)	134 (78)	< 0.001
Posterolateral	153 (77)	39 (22)	
Indication^b^			
Primary	157 (79)	152 (88)	0.02
Secondary	42 (21)	21 (12)	
Type of arthroplasty **^b^**			
HA	104 (52)	116 (67)	0.004
Cemented THA	44 (22)	44 (25)	
Reversed hybrid THA	51 (26)	13 (8)	
Surgeons experience **^b^**			
Consultant	195 (98)	162 (93)	0.03
Registrar	4 (2)	11 (7)	

**^a^** mean (SD)

**^b^** n (%)

We collected data on all hips, including indication for surgery (primary or secondary surgery), type of arthroplasty (HA, cemented THA, or reversed hybrid THA), surgical approach, surgery time (skin-to-skin), experience of the surgeon (consultant or registrar), cognitive dysfunction (no/probable/certain), dislocations, and all complications leading to reoperation. All patients were followed up at 3 months postoperatively. Furthermore, a search of our medical database was carried out until Aug 2009 to identify any dislocations or reoperations. The Swedish death register was used to verify mortality. We used the Swedish Hip Joint Register to search for patients who had undergone reoperation elsewhere in Sweden. No such cases were found.

At our orthopedics department, hip arthroplasty is performed on patients with a displaced femoral neck fracture who are 65 years old or more and who have previously been independent walkers (with or without walking aids). Patients 80 years and over are normally treated with an HA, but the decision of whether to perform an HA or a THA is done according to the surgeon's preference and the patient's level of activity.

From 2008, a policy change was implemented regarding surgical approach and it was recommended that all surgeons should use the anterolateral approach when performing hip arthroplasty on patients with a femoral neck fracture. To study the consequences of this change, we divided all hips into 2 groups, hips operated during 2007 and those operated during 2008. However, during both time periods the individual surgeons were free to choose whether to use a posterolateral approach (Moore 1957) with repair of the posterior capsule and external rotators (Kwon et al. 2006), or an anterolateral approach with the patient in the lateral decubitus position (Gammer 1985). We did not formalize the training of the surgeons for use of the anterolateral approach.

During the study, 27 surgeons performed median 12 (1–43) arthroplasties. 4 surgeons had had previous experience of using the anterolateral approach before the start of the study, and they assisted the less experienced surgeons if required. The patients were operated with a cemented polished tapered femoral stem (CPT; Zimmer, Warsaw, IN) or an uncemented tapered femoral stem (Bi-Metric; Biomet, Warsaw, IN). A cemented cup was used routinely (ZCA; Zimmer). When performing an HA, the cemented stem was used in all cases and combined with a unipolar Cr-Co head. A 32-mm Cr-Co head was used when performing a THA. Thus, three types of arthroplasties were performed: HA, cemented THA, and reversed hybrid THA. All patients received prophylactic antibiotics intravenously during the first 24 h postoperatively and daltaperin (Fragmin; Pharmacia, Sweden) postoperatively until fully mobilized. The patients were allowed full weight bearing. Crutches were used for support and the patients were mobilized in accordance with a standard physiotherapy program. No braces were used to avoid dislocations.

Following the change to the new recommendation, the rate of hip operations performed with the anterolateral approach rose from 23% in 2007 to 78% in 2008. There were no clinically significant differences regarding age, sex, weight, height, ASA category, or cognitive dysfunction between the 2007 and 2008 groups. However, in 2008 there were more arthroplasties performed as a primary intervention, there were less reversed hybrid THAs performed, and more of the arthroplasties were performed by registrars.

The study was done in accordance with the ethical principles of the Helsinki declaration. The Ethics Committee of the Karolinska Institute approved the study.

### Statistics

Student's t-test and the chi-squared test were used to detect differences between the 2 time periods regarding anthropometric and surgical data. A multivariate logistic regression analysis was used to analyze differences between the 2 years and to adjust for potential confounding factors. The demographic and implant factors used to evaluate which factors increased the risk of dislocation were sex, age at surgery, ASA category, cognitive dysfunction, indication for surgery, type of arthroplasty, the surgeon's experience, and the surgical approach. The data are presented as odds ratios (ORs). All results were considered significant at p < 0.05. The statistical analysis was performed using PASW Statistics software for Windows (SPSS Inc., Chicago, IL).

## Results

19 (5%) of the 372 hips in the study dislocated at least once during the study period (January 2007 to August 2009). Dislocations occurred at a median of 13 (1–85) days postoperatively. The dislocation rate was 8% (16/199) for patients who underwent arthroplasty during 2007 and 2% (3/173) for those in 2008 (p = 0.006). 14 of the 16 hips that dislocated in 2007 were operated with the posterolateral approach. The 3 dislocating hips from the 2008 group were all operated with the posterolateral approach. Overall, the dislocation rate for the whole study period was 9% (17/192) for the posterolateral approach and 1% (2/180) for the anterolateral approach (Table 2).

**Table 2.** Details on all 19 hips in the study that dislocated

A	B	C	D	E	F	G	H	I	J	K
1	83	F	2	No	2007	1	Primary	Anterolateral	HA	Excision arthroplasty after multiple dislocations
2	84	M	2	No	2007	5	Secondary	Anterolateral	Cemented THA	Excision arthroplasty after multiple dislocations
3	60	M	3	No	2007	3	Secondary	Posterolateral	Reversed hybrid THA	One dislocation, closed reduction
4	67	F	2	No	2007	4	Primary	Posterolateral	Cemented THA	One dislocation, closed reduction
5	73	M	2	No	2007	13	Primary	Posterolateral	Reversed hybrid THA	Constrained cup after multiple dislocations
6	75	M	3	Probable	2007	16	Primary	Posterolateral	HA	Excision arthroplasty after multiple dislocations
7	76	F	3	No	2007	2	Primary	Posterolateral	Cemented THA	Multiple dislocations and closed reductions
8	76	M	2	No	2007	32	Primary	Posterolateral	Reversed hybrid THA	Multiple dislocations and closed reductions
9	77	F	2	No	2007	15	Primary	Posterolateral	Reversed hybrid THA	One dislocation, closed reduction
10	80	F	3	No	2007	1	Primary	Posterolateral	HA	One dislocation, open reduction and stem revision
11	81	F	3	No	2007	1	Primary	Posterolateral	HA	One dislocation, open reduction
12	82	F	3	No	2007	76	Primary	Posterolateral	Cemented THA	One dislocation, closed reduction
13	82	F	2	No	2007	38	Primary	Posterolateral	Reversed hybrid THA	One dislocation, closed reduction
14	83	F	3	No	2007	1	Secondary	Posterolateral	HA	Excision arthroplasty after multiple dislocations
15	84	F	3	No	2007	54	Primary	Posterolateral	HA	One dislocation, closed reduction
16	89	F	3	No	2007	11	Secondary	Posterolateral	Reversed hybrid THA	One dislocation, closed reduction
17	67	F	2	No	2008	70	Primary	Posterolateral	Cemented THA	One dislocation, closed reduction
18	70	F	2	Certain	2008	85	Primary	Posterolateral	Cemented THA	Constrained cup after multiple dislocations
19	88	F	2	Probable	2008	15	Primary	Posterolateral	HA	Multiple dislocations and closed reductions

A No. B Age C Sex D ASA class E Cognitive dysfunction F Year G Time to first dislocation (days) H Primary or secondary surgery I Surgical approach J Type of arthroplasty K Final outcome

In the multivariate logistic regression analysis, the posterolateral approach was the only factor associated with a significantly increased risk of dislocation, with an OR of 8 (2–35). Age, sex, ASA category, cognitive dysfunction, the indication for surgery, the type of arthroplasty, and the experience of the surgeon had no effect on the dislocation rate (Table 3 and Figure).

**Table 3.** Multivariate logistic regression to evaluate factors associated with dislocation

Explanatory	n (372)	Dislocation rate (%)	OR (95% CI)	p-value
Age				
≤ 82	194	6.7	1	
> 82	178	3.4	0.7 (0.2–1.9)	0.5
Sex				
Male	95	5.3	1	
Female	277	5.1	1.1 (0.4–3.1)	0.8
ASA-category				
ASA 1–2	186	5.4	1	
ASA 3–4	186	4.8	1.1 (0.4–3.1)	0.8
Cognitive dysfunction				
No	288	5.9	1	
Probable	33	3.0	0.7 (0.1–6.2)	0.7
Certain	51	2.0	0.4 (0.1–3.4)	0.4
Indication				
Primary	309	4.9	1	
Secondary	63	6.3	1.0 (0.3–3.4)	1.0
Type of arthroplasty				
HA	220	3.2	1	
Cemented THA	88	6.8	1.6 (0.4–5.9)	0.5
Reversed hybrid THA	64	9.4	1.4 (0.4–5.0)	0.6
Surgeon's experience				
Consultant	357	5.3	1	
Registrar	15	0.0	0.2 (0.0–4.1)	1.0
Surgical approach				
Anterolateral	180	1.1	1	
Posterolateral	192	8.9	7.6 (1.7–34.8)	0.01

**Figure:**
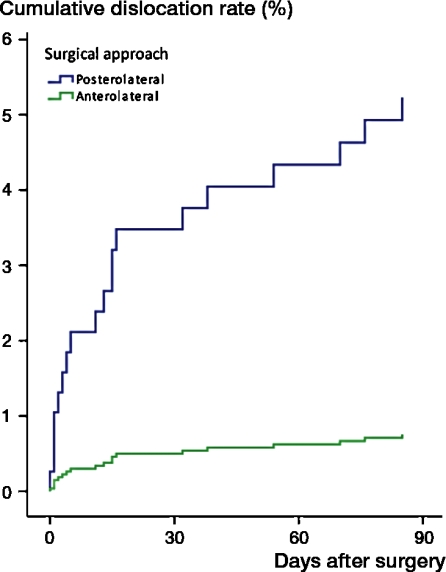
Cox regression cumulative dislocation rate adjusted for sex, age, cognitive function, and type of arthroplasty.

35 patients (18%) in the 2007 group and 20 (12%) in the 2008 group died during the study period. There was no statistically significant difference between the 2 time periods regarding mortality when we adjusted for the different follow-up times (p = 0.8, log-rank test). The 2 groups were also similar regarding complications leading to reoperation (Table 4), medical complications after surgery (data not shown), and mean length of surgery (81 (SD 28) and 84 (SD 24) min for 2007 and 2008, respectively).

**Table 4.** Numbers of complications leading to reoperation

Complication	2007 (n = 199)	2008 (n = 173)
Deep infection	5	2
Periprosthetic fracture	3	4
Early aseptic loosening	1	0

## Discussion

We have shown that it is possible to reduce the dislocation rate dramatically for a whole orthopedics department by changing the surgical approach used for hip arthroplasty in patients with femoral neck fractures. To avoid selection bias during the 2 years of the study, we included all patients regardless of surgical approach. This report therefore reflects the whole department's learning curve of the anterolateral approach.

Except for dislocation rate, we have not taken into consideration any other aspects of the different approaches studied. It has been suggested that differences exist between the two approaches in terms of types of complications such as nerve injuries (Ramesh et al. 1996, Picado et al. 2007), gluteus medius insufficiency (Baker and Bitounis 1989), trochanteritis and rupture of the medius or external rotator repair. All of these factors can influence the patient's hip function and quality of life, and these are important parameters that we have not been able to assess in this study. Furthermore, surgical access to the acetabulum and femoral canal may vary between the two approaches and influence the surgical outcome. In the last report from the Swedish Hip Arthroplasty Register, the posterolateral approach was associated with a lower revision rate due to aseptic loosening and also higher patient satisfaction compared to the anterolateral approach. However, both for HA in patients with femoral neck fractures and THA in all patients, the anterolateral approach was associated with a lower risk of revision due to dislocation compared to the posterolateral approach (Garellick et al. 2009).

A dislocation can have dire consequences for the patient involved, particularly for elderly patients with a femoral neck fracture and multiple comorbidities. Of the 19 patients with dislocations during the study, 3 have undergone implant revision, 4 were converted to excision arthroplasty, and 3 have experienced more dislocations. The alternatives for revision surgery on a dislocating hip such as cup revision, stem revision, or changing to a larger articulation is often not feasible because of comorbidities. Thus, the consequences for the dislocating hip fracture patient can be to live with a severely debilitating excision arthroplasty or lowered quality of life due to a series of dislocations (Enocson et al. 2009b). For hip fracture patients, the primary intervention is crucial and all our efforts should aim for a “first time right”.

All posterolateral approaches in this study were done with repair of the posterior capsule and external rotators. Despite this, we had an overall dislocation rate of 9% for the posterolateral approach. Kwon et al. (2006) lowered the dislocation rate in patients with osteoarthritis of the hip from 4% to 0.5% by repairing the capsule and external rotators. In two recently published studies on patients with a femoral neck fracture, the dislocation rate for posterolateral approach with and without posterior repair was 9% and 13%, respectively, for HAs and 12% and 14% for THAs (Enocson et al. 2008, 2009a). Thus, the posterior repair appears to give only marginal protection against dislocation in patients with femoral neck fractures, possibly because of traumatized and weakened posterior structures.

Our study has several limitations. The selection differed somewhat between the two different years. There were more reversed hybrid THAs performed in 2007, more HAs in 2008, and more secondary procedures in 2007. All these factors could possibly have contributed to the higher dislocation rate in 2007. When adjusting for these inequalities with Cox regression, none of these factors were found to have an impact on the result except the anterolateral approach. The number of patients included differed between the 2 years: 199 in 2007 and 173 in 2008. The reason was not a change in indication for arthroplasty but rather a random change in the number of patients. The rate of arthroplasties performed at our department on patients with a femoral neck fracture was the same for both years (data not shown). Since we scrutinized all the medical records up until August 2009, the follow-up time differed between the patients who underwent surgery in 2007 and 2008. However, all dislocations and other complications leading to reoperations occurred within the first 3 months after surgery. Thus, the difference in follow-up time should not have influenced our results. In contrast to other reports (Johansson et al. 2000), cognitive function did not appear to have adversely affected the dislocation rate. However, no validated score to assess cognitive function was used, apart from the surgeon's subjective assessment of the patient.

In conclusion, dislocation is one of the greatest threats to full recovery and the return to well-being for the patient undergoing hip arthroplasty as a result of femoral neck fracture. We believe that a collected policy change regarding surgical approach for these patients is feasible and to be recommended, as it leads to a substantial reduction in dislocation rate.
